# Impact of SARS-CoV-2 Virus (COVID-19) Preventative Measures on Communication: A Scoping Review

**DOI:** 10.3389/fpubh.2022.815259

**Published:** 2022-03-28

**Authors:** Ilze Oosthuizen, Gabrielle H. Saunders, Vinaya Manchaiah, De Wet Swanepoel

**Affiliations:** ^1^Department of Speech-language Pathology and Audiology, University of Pretoria, Pretoria, South Africa; ^2^Virtual Hearing Lab, Collaborative Initiative Between University of Colorado School of Medicine, Aurora, CO, United States, and University of Pretoria, Pretoria, South Africa; ^3^Manchester Centre for Audiology and Deafness (ManCAD), University of Manchester, Manchester, United Kingdom; ^4^Department of Otolaryngology–Head and Neck Surgery, University of Colorado School of Medicine, Aurora, CO, United States; ^5^UCHealth Hearing and Balance, University of Colorado Hospital, Aurora, CO, United States; ^6^Department of Speech and Hearing, School of Allied Health Sciences, Manipal Academy of Higher Education, Manipal, India; ^7^Ear Science Institute Australia, Subiaco, WA, Australia

**Keywords:** preventative measures, face masks, distancing, COVID-19, communication

## Abstract

**Introduction:**

Face coverings and distancing as preventative measures against the spread of the Coronavirus disease 2019 may impact communication in several ways that may disproportionately affect people with hearing loss. A scoping review was conducted to examine existing literature on the impact of preventative measures on communication and to characterize the clinical implications.

**Method:**

A systematic search of three electronic databases (Scopus, PubMed, CINAHL) was conducted yielding 2,158 articles. After removing duplicates and screening to determine inclusion eligibility, key data were extracted from the 50 included articles. Findings are reported following the Preferred Reporting Items for Systematic Reviews and Meta-analyses (PRISMA) Extension for Scoping Reviews, including the PRISMA-ScR checklist.

**Results:**

Studies fell into three categories: Studies addressing the impacts of personal protective equipment (PPE) and/or distancing on communication in healthcare contexts (*n* = 20); studies examining the impact of preventative measures on communication in everyday life (*n* = 13), and studies measuring the impact of face coverings on speech using acoustic and/or behavioral measures (*n* = 29). The review revealed that masks disrupt verbal and non-verbal communication, as well as emotional and social wellbeing and they impact people with hearing loss more than those without. These findings are presumably because opaque masks attenuate sound at frequencies above 1 kHz, and conceal the mouth and lips making lipreading impossible, and limit visibility of facial expressions. While surgical masks cause relatively little sound attenuation, transparent masks and face shields are highly attenuating. However, they are preferred by people with hearing loss because they give access to visual cues.

**Conclusion:**

Face coverings and social distancing has detrimental effects that extend well beyond verbal and non-verbal communication, by affecting wellbeing and quality of life. As these measures will likely be part of everyday life for the foreseeable future, we propose that it is necessary to support effective communication, especially in healthcare settings and for people with hearing loss.

## Introduction

The World Health Organization (WHO) declared the Coronavirus disease 2019 (COVID-19) a pandemic on March 11, 2020 (1). In response, the WHO and many governments around the world rapidly developed guidelines about use of personal protective equipment (PPE) and other measures (e.g., physical/social distancing) to decrease the spread of the virus. Although details of the guidelines varied across the globe, almost all countries recommended that face coverings (masks) be worn indoors and/or when in close proximity to others. While lowering the risk of infection (2), these measures can disrupt communication by altering transmission of the acoustical signal, preventing lipreading, limiting the interpretation of facial expressions, and changing social cues and nuances (3–9). Published studies examining the impacts of preventative measures (i.e., PPE/face coverings and/or distancing) on communication have been conducted using a variety of approaches, as reviewed below. These included acoustic measures of sound transmissions through masks, performance-based testing of speech understanding and face recognition, and surveys of the general public, healthcare professionals and patients. Many studies have focused on the impacts of preventive measures on communication in health care settings, some examined healthcare professional (HCP)-HCP communication, while others have addressed HCP-to-patient communication.

People with hearing loss or who are deaf are particularly vulnerable to communication problems associated with use of preventative measures. Given the global burden of hearing loss, affecting more than 20% of the global population (10, 11), consideration of the impact of preventative measures on communication for people with hearing loss is a priority. To date however only a handful of studies have directly addressed this (6, 12–14). While transparent face masks have been proposed as a potential solution to alleviate communication problems associated with covering the face and lips (6), data suggest that the materials used in transparent face masks are acoustically more attenuating than materials used in non-transparent masks (3, 15), thus potentially negating their benefit.

With the ongoing COVID-19 pandemic and proliferation of publications examining the impact of face coverings and other preventive measures on communication, there is a need for a review to identify and document the extent, range and nature of findings about this. We therefore conducted this scoping review examining the extant literature on the impacts of preventative measures on communication with a view to understand how PPE and distancing impact communication and to characterize the clinical implications. A scoping review is appropriate because the topic is exploratory and broad (16) and because it is suited to identify and map the available evidence (17, 18).

## Methods

This scoping review was conducted using published guidelines (16, 17, 19) and the Preferred Reporting Items for Systematic Reviews and Meta-Analyses Extension for Scoping Reviews (PRISMA-ScR) checklist (20) (Supplementary Table 1). Institutional review board approval was granted for this study by the Research Ethics Committee of the Faculty of Humanities, University of Pretoria.

### Study Purpose

The purpose of this study was to examine the extent, range, and nature of research concerning the impacts of PPE and physical/social distancing against the spread of COVID-19 on communication by triangulating findings from acoustic, performance-based and survey measures. The ultimate goal is to identify the various impacts that PPE and distancing can have on communication and to characterize the clinical implications of the findings.

### Search Strategy and Study Selection

Three databases (i.e., Scopus, PubMed, and CINAHL) were searched for relevant literature from their inception until the date the search was concluded (01 September 2021). Key search terms used were “hearing,” “hearing loss,” “hearing impairment,” “hearing diff^*^,” “deaf,” “hearing ability,” “communication,” “communication diff^*^” combined with “personal protection,” “PPE,” “masks,” “face covering^*^,” “physical distanc^*^,” “social distanc^*^.” Electronic search results were exported to Rayyan software (https://www.rayyan.ai/) (21) and duplicates deleted. The search yielded 2,158 articles, of which 376 duplicates were deleted, leaving 1,782 unique references. The Rayyan software was used to screen these unique references and to record decisions about inclusion. The initial screening was based on the content of the title and abstract. A full text review followed. Papers were included if they met the following criteria: (a) primary research study published in a peer-reviewed journal; (b) impacts of PPE and distancing on communication were assessed in some manner, and (c) the full-text article was available in English. Studies focusing on audiological and/or vestibular symptoms (e.g., hearing loss and/or tinnitus) arising from COVID-19 and studies examining the impact of respirators and alternative experimental face coverings were excluded. Authors IO and GS screened the studies independently. Discrepancies were then discussed. When further resolution was required, as in the case of four studies, authors VM and DS made a final decision.

### Data Extraction

Guidelines for data extraction were developed jointly by the study authors. Authors IO and GS then reviewed two articles independently and compared their results. This allowed assurance that their interpretation of the guidelines was identical and led to refinement of the extraction guidelines. The remaining articles were subsequently distributed amongst all authors, who independently extracted and tabulated the data in a Microsoft Excel spreadsheet. Information extracted were study authors, title, publication year, country of first author and country of participants, study aims relating to the impact of preventative measures on communication, study context relative to COVID-19, sample description, data collection methods, variables assessed, summary of key findings and the author's interpreted clinical implications.

### Synthesis of Results

The findings were synthesized with input from all authors with the goals of identifying (a) the various impacts that PPE and distancing can have on communication and (b) the clinical implications of the findings. Results are presented descriptively due to the heterogeneity of studies and the scoping nature of the review.

## Results

### Search Results

**Figure 1:**
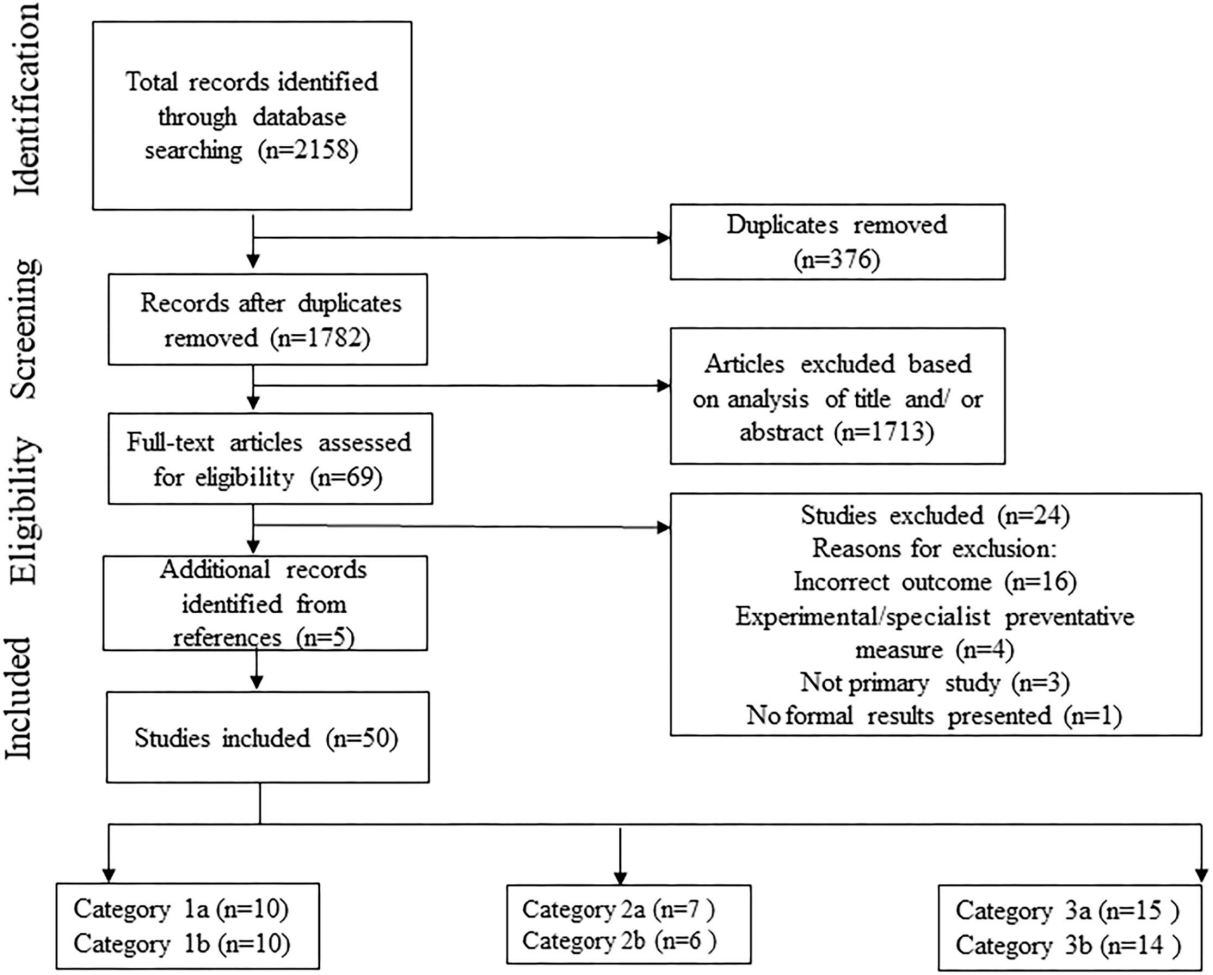
PRISMA flow diagram that details the search and selection process applied during the scoping review. Sum of studies from subcategories >50 as some studies addressed more than one subcategory.

From the 1,782 unique records identified in the searches, 45 studies were deemed appropriate for inclusion. Following a hand-search of references of the included articles, an additional five studies were identified resulting in a total of 50 studies. Further details are shown in Figure 1.

The included studies were classified into three categories, each of which had two subcategories, as follows:

1. Impacts of PPE and/or Distancing on Communication in Healthcare Context.
  1a. HCP-HCP communication.
  1b. HCP-patient communication.
2. Impacts of Face Coverings and/or Distancing on Communication in Everyday Life.
  2a. For the general public.
  2b. For people with hearing loss.
3. Impacts of Face Coverings (i.e., Face Masks, Face Shields etc.) on Speech.
  3a. Assessed via acoustic measures (i.e., objective measurements of sound).
  3b. Assessed via behavioral measures (i.e., performance tests conducted by participants).

**Figure 2:**
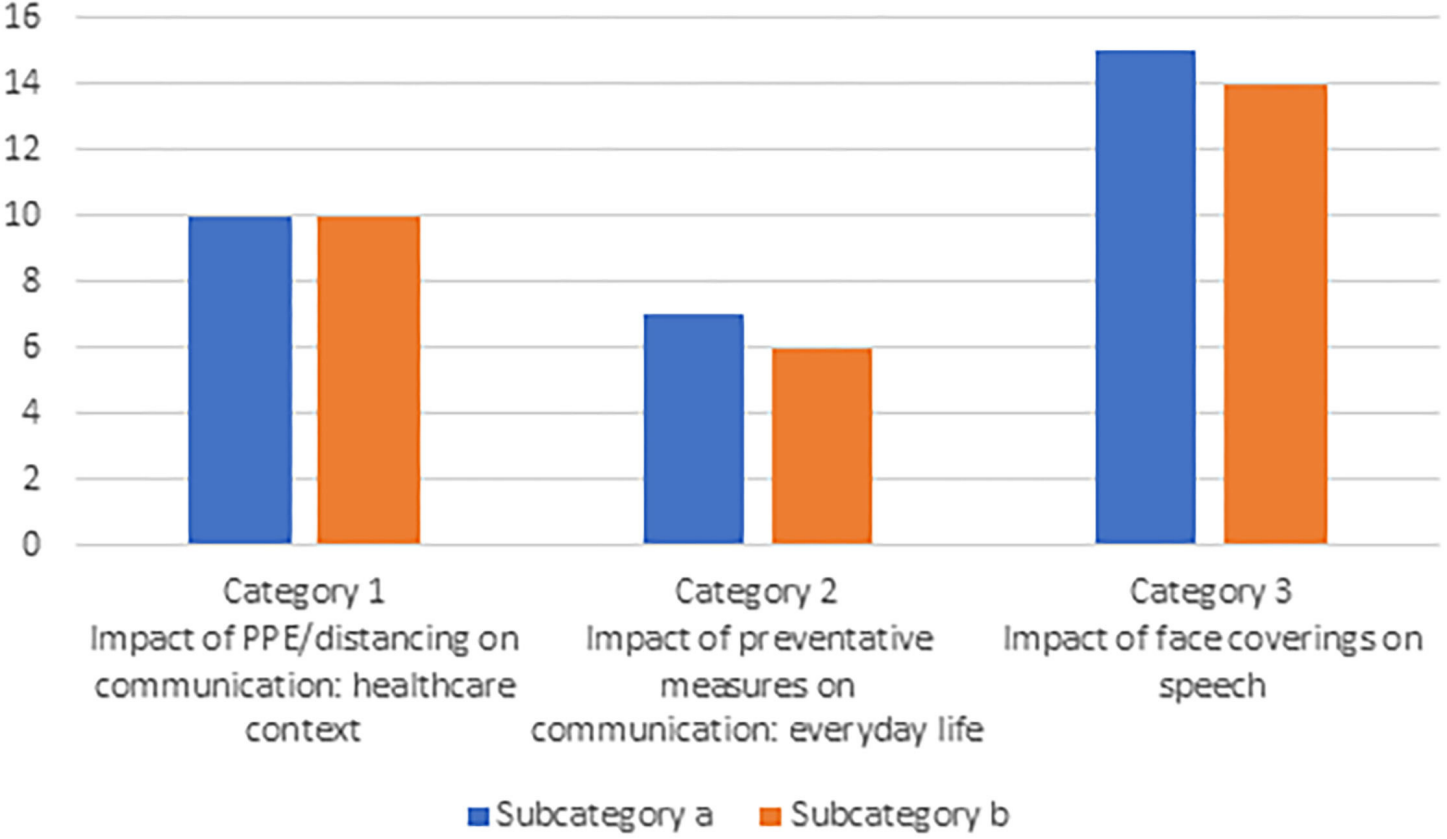
Studies included in the scoping review per category and subcategory.

Figure 2 shows a breakdown of the number of articles by subcategory.

A summary of the findings is presented in Table 1 and is described below by category. The data extracted from each paper is summarized in Supplementary Tables 2–4.

**Table 1:** Summary of studies on the impact of COVID-19 preventative measures on communication included in scoping review (*n* = 50) according to study subcategory.

	**No. studies**	**Methodologies**	**Summary of findings**
**1. Impacts of PPE and/or distancing on communication in healthcare context**			
1a. HCP-HCP communication	10	Surveys (*n* = 8), semi-structured interviews (*n* = 1), combined interviews and media reports (*n* = 1)	Masks primarily disrupt speech communication, but also affect interpersonal relationships and face recognition. Transparent masks were typically unavailable.
1b. HCP-patient communication	10	Surveys (*n* = 8), semi-structured interviews (*n* = 1), combined interviews and media reports (*n* = 1)	Impacts on both verbal (speech understanding) and non-verbal communication including trust and rapport. Patients feel anxious and fearful. Transparent masks had positive impacts on outcome
**2. Impacts of face coverings and/or distancing on communication in everyday life**			
2a. For the general public	7	Behavioral measures (*n* = 4), surveys (*n* = 3)	Masks have broad psychosocial impacts as well as negatively impacting hearing and communication.
2b. For people with hearing loss	6	Surveys (*n* = 6)	Communication difficulties due to hearing loss are exacerbated by masks, impacting quality of life and wellbeing. People with hearing loss prefer transparent masks over opaque masks.
**3. Impacts of face coverings (i.e., face masks, face shields etc.) on speech**			
3a. Assessed via acoustic measures	15	Combinations of measured attenuation (*n* = 9), speech indices (*n* = 3), spectral analyses (*n* = 3), directional analyses (*n* = 2), and voice parameters (*n* = 3)	Masks attenuate sound above 1 kHz. Surgical masks cause least attenuation; transparent masks and face shields the most. Transparent masks/shield affect directionality. Perceived vocal effort is increased.
3b. Assessed via behavioral measures	14	Combinations of speech testing in quiet (*n* = 7) and in noise (*n* = 10) for auditory alone (*n* = 8) and auditory-visual (*n* = 6) conditions	Face coverings decreased speech scores but was dependent on type, listening condition and hearing status. Transparent masks were relatively more beneficial to people with hearing loss than to people without hearing loss.

### Impacts of PPE and/or Distancing on Communication in Healthcare Context: HCP-HCP Communication

Ten studies investigated the impact of PPE and/or distancing on communication between HCPs. Eight studies used surveys (22–29), one used semi-structured online interviews (30), and one combined information from semi-structured phone interviews, media reports and government policies (31). Broadly, the surveys and semi-structured interviews indicated that disrupted speech communication between HCPs is among the most common problems arising from use of PPE in a medical context. One study that focused on hearing loss showed that use of opaque face masks resulted in HCPs who are D/deaf feeling fearful of making errors during their medical practice, and that the level of anxiety reported was strongly associated with degree of hearing loss (26). Almost none of the respondents worked in a location that had access to transparent masks (26). The two studies that explored the impact of social distancing in combination with use of PPE indicated that both negatively affected communication and interpersonal relationships in healthcare contexts (24, 30). Finally, two studies reported that opaque face masks lead to difficulties recognizing colleagues (30, 31).

### Impacts of PPE and/or Distancing on Communication in Healthcare Context: HCP-Patient Communication

Ten studies explored how PPE and/or distancing affected communication between HCPs and patients. With the exception of two, all were survey-based studies (24, 26, 32–37). The two exceptions used semi-structured interviews, with one supplementing this with information from media reports and government policies (31, 38). Nine of the ten studies reported that PPE and/or distancing caused barriers to effective verbal and non-verbal communication with patients, especially for HCPs who are D/deaf (26), and that it negatively impacted psychosocial factors such as trust and rapport, and resulted in patients (including pediatric patients) feeling fearful/anxious during appointments. A study by Kratzke et al. (36) revealed that using transparent masks instead of opaque masks had positive impacts on HCP-patient interactions, leading to improved understanding and increased trust and perceived empathy. Two studies addressed communication between HCPs and patients with hearing loss. Results indicated that HCPs had become more aware of communication difficulties associated with hearing loss since the start of the pandemic. This was attributed to face masks muffling speech, preventing lipreading, and making encounters longer (31, 32).

### Impacts of Face Coverings and/or Distancing on Communication in Everyday Life for the General Public

Seven studies investigated the impacts of face coverings and/or distancing on communication in everyday life for the general public. Four of these studies used behavioral measures (7, 8, 39, 40) and three used surveys (12, 41, 42). Self-reported difficulties from the surveys showed that the effects of face masks and/or distancing extended beyond hearing and communication difficulties to general health, quality of life, and psychosocial state/functioning. The four studies that used behavioral measures determined that face masks decreased the ability to recognize emotions, reduced perceived interpersonal closeness, and decreased facial mimicry during communication. Further, when masks were used during oral examinations, students perceived their test performance to be negatively impacted, although in fact, test scores were unaffected (40).

### Impacts of Face Coverings and/or Distancing on Communication in Everyday Life for People With Hearing Loss

Six survey-based studies, revealing self-reported communication difficulties experienced by people with hearing loss, were identified in this subcategory. Three studies examined the impacts of face coverings and/or social distancing on general communication. They indicated masks and social distancing exacerbated the communication difficulties people with hearing loss already experience, and that this negatively affects quality of life, willingness to engage in social interactions, and leads to emotional and psychosocial challenges (12, 43, 44). Studies also showed that challenges arise in healthcare and education settings (12, 45), as well as during virtual communication (43). One study showed the impacts of preventive measures to be significantly greater for people with hearing loss than for those without (12), and another reported on the practical issues that arise from wearing hearing aids with a mask (46). People with hearing loss indicated a preference for HCPs to use transparent masks during appointments (44), and despite communication problems, most hearing aid users prioritized their hearing health as reflected in their attending audiology appointments (46). Interestingly, two studies reported that distancing measures impacted communication positively for people with hearing loss, because communication was taking place in quieter situations, with consequent improved speech understanding and less perceived listening effort (44, 47).

### Impacts of Face Coverings on Speech Assessed *via* Acoustic Measures

Fifteen studies used acoustic measurements to assess the impact of various face coverings on transmission of sound (3, 6, 9, 13–15, 48–56). The results showed that, to varying extents, masks and face shields attenuated the transmission of sound frequencies above 1 kHz. Transparent plastic face shields attenuated sounds the most (49, 51), surgical masks the least (3, 6, 9, 15, 48, 55). Generally, transparent face masks, N95 masks, and cloth masks fell somewhere between (3, 9, 15, 48, 52, 55). The attenuating effect of a cloth mask depended on the type and weave of the cloth and on its fit, with masks made from breathable fabrics such as cotton and masks with pleats, having better acoustic performance than non-breathable, non-pleated masks (3, 55). Not surprisingly, the attenuating effect of wearing a face mask in combination with a face shield was cumulative (49, 54, 56). The directionality of sound attenuation was assessed by two studies which showed that attenuation was the greatest toward the front for all masks, and that plastic face shields deflect and amplify sound behind the talker (3, 15). Finally, three studies investigated the impact of face masks on voice/vocal effort used when wearing a mask (50, 52, 53). These studies showed masks did not change the acoustical properties of the voice. However, participants reported decreased vocal intensity and increased vocal effort and dyspnea when speaking wearing a mask.

### Impacts of Face Coverings on Speech Assessed *via* Behavioral Measures

Fourteen studies used performance-based speech tests to explore the effect of face coverings on speech understanding in quiet and/or in noise (6, 13, 14, 48, 51, 52, 54–61). In general, the results showed the same as those of the acoustic measures, namely that face coverings had a detrimental effect on speech understanding. However, in some instances, the decrement in the acoustic signal did not translate into decreased speech understanding scores. As further described below, the impact face coverings had on speech understanding depended on the type of face covering, the listening condition (quiet vs. noise) and the hearing status of the listener. With just two exceptions (54, 59), studies showed that surgical masks had no impact on speech understanding in quiet for people with normal hearing (52, 61) and little or no impact on speech understanding in noise (6, 13, 48, 55). Cloth/fabric masks and N95 masks, on the other hand, did affect speech understanding in noise for people with normal hearing (48, 55), as did the combination of a face mask and a face shield (54, 57). Five studies included people with hearing loss (6, 13, 14, 51, 56). Three of these showed masks to be detrimental to speech understanding (6, 14, 51), and three showed speech understanding to be improved with a transparent mask/face shield relative to an opaque mask (6, 14, 51). The combination of a face mask plus face shield also resulted in decreased speech understanding in quiet for cochlear implant users relative to a no-mask or mask-only condition (56).

Three studies combined behavioral testing with quantitative self-report questions. They showed that opaque masks resulted in subjective difficulties, the need for increased concentration/listening effort, and decreased confidence in understanding relative to no mask and/or transparent masks. This was the case regardless of hearing status (14, 48, 59). In addition, two studies examined the interaction between face masks and speech style (58, 61). Results indicated using a clear speaking style when wearing a mask resulted in improved speech understanding for people with normal hearing relative to a masked conversational speaking style and no mask conditions. One study revealed that PPE affected speech understanding in simulated hospital environments with high background noise levels (60).

## Discussion

This scoping review reports research examining the impact of COVID-19 preventative measures on communication, to identify the various impacts that PPE and distancing can have on communication and to characterize the clinical implications. Fifty empirical studies were included, with the majority published since the start of the pandemic in 2020 when the wearing of face coverings became mandatory or strongly recommended around the world. The key findings are discussed below.

The detrimental impact of face coverings on communication broadly is evident across the published research. From an acoustic perspective, face coverings reduce transmission of the speech signal at frequencies above 1 kHz which has consequences for hearing the speech signal (3, 15). In some studies this is reflected in poorer performance on speech understanding tests (14, 48), but not always (13, 55, 61). It depends on the material of the mask, the listening conditions and hearing status. Specifically, surgical masks have little impact on speech understanding performance, cloth/fabric masks, face shields and/or mask-face shield combinations have more; speech in noise is more affected than speech in quiet; and people with hearing loss are more affected than people with normal hearing. The picture is more complex when considering transparent masks in that while they attenuate sound more than opaque masks (3, 6, 15, 56), people with hearing loss benefit from their use (6, 14). This is presumably because the mouth and lips are visible allowing for use of lipreading and other non-verbal cues (6, 14).

These findings are echoed in survey responses, with participants reporting that masks muffle and attenuate speech, and people with hearing loss reporting more difficulties than those without (22, 33, 42, 44). However, acoustic and behavioral measures do not capture the psychosocial effects that opaque masks have on communication in terms of limiting ability to recognize emotions from facial expressions, decreasing feelings of engagement in a conversation and emotional connection with the speaker and self-confidence (7, 12, 14, 40, 42, 59), eliciting feelings of loneliness, depression, fear, and anxiety (12, 43, 44). Surveys also reveal that masks lead to a need to concentrate harder and use more effort during communication (14, 48, 59), and thus increase listening-related fatigue. Although broadly reported, the impacts are significantly greater for people with hearing loss than for those with normal hearing (12, 43, 44).

Masks have always been a part of medical practice, however, their now ubiquitous use has had broad impacts on communication within the medical context. In addition to detrimental effects on verbal interactions, masks have been reported to affect HCP-HCP and HCP-patient interpersonal relationships, decrease trust and perceived empathy, create fear and anxiety in patient, and reduce HCP's situational awareness-all of which can subsequently affect quality of care (24, 28, 30, 31, 33, 34, 36, 38). Indeed, Trecca and colleagues (62) reported that more patients with hearing loss attributed problems with surgical masks to the inability to lip read than to the speech being muffled, while the study of Kratzke et al. (36) found patients perceived HCPs to be more empathetic and trustworthy than when they are wearing transparent masks rather than opaque masks. The impact of masks in a medical settings is particularly negative for elderly persons, people with hearing loss (12, 31, 32), and for HCPs with hearing loss (26).

Studies showed that the negative impacts of face coverings on communication were more pronounced in noise than in quiet. Unfortunately, educational settings are situations in which communication is critical, masks are now widely worn, and noise levels are typically high. School-age children with hearing loss may be particularly vulnerable to the impact of non-transparent face masks used by teachers (45), especially if they are still developing language and need optimal access to communication models and to visual cues (63).

The impacts of distancing measures on communication were considered in just a few studies. They showed that distancing compounds the negative effects of masking on audibility and communication, in everyday life (42, 43), and in educational contexts, especially for students with hearing loss (45). This is presumably because increased distance between a speaker and a listener leads to a decrease in signal to noise ratio, thus affecting audibility and speech understanding. However, it is noteworthy that people with hearing loss reported a positive impact of distancing measures on their communication as it resulted in spending more time in favorable (quieter) listening environments with consequent better speech understanding and reduced listening effort (44, 47). In fact, one study found COVID-19 distancing measures reduced environmental sounds levels by ~3 dBA (64)-which is enough to be beneficial to hearing for all people (64).

### Clinical and Educational Implications

The findings of this review point to a number of clinical and educational implications as summarized in Table 2.

**Table 2:** Clinical and educational implications from scoping review: key recommendations to lessen the impact of COVID-19 preventative measures on communication.

**Recommendation**	**Motivation**
**Reduce background noise**	
Healthcare contexts Educational environments Communicating with people with hearing loss	Improve speech understanding Reduce anxiety to hear
**Type of mask Transparent masks**	
Especially when communicating with people with hearing loss	Access to visual cues / speechreading cues improve speech understanding Reduce listening effort Visibility of emotions from facial expressions help improve empathy and trust
**Opaque masks**	
Surgical mask or N95 mask	Less impact on speech signal and speech understanding than other opaque masks
**Use remote microphone technology**	
Classroom settings Communicating with people with hearing loss	Improve speech understanding
**Use augmentative strategies**	
Speak slowly and clearly Use gestures Do no shout Rephrase rather than repeat verbatim Write key information	Improve communication interaction overall
**Use of remote or virtual communication**	
Quiet environment with good lighting Look directly at camera Use live subtitling	Improve clear communication; take care to ensure empathetic communication

- Reduce background noise where possible, especially in situations where important information is conveyed (e.g., healthcare and education contexts) to support better speech understanding and relieve anxiety around hearing. This can be achieved through environmental modifications (e.g., use soft furnishings) and turning off extraneous sound sources (e.g., radio, TV).
- Select the type of mask based on communication needs. Transparent masks improve speech understanding for people with hearing loss when visual cues are available (6, 14). However, they are more acoustically attenuating than opaque masks, therefore they are not recommended in situations when visual cues are absent or are less important. Research on face shields is limited, but although they provide the best visibility of the face, they are highly attenuating, particularly from signals originating from the front (see below), thus recommendations about their use cannot currently be made. If an opaque mask is to be used, surgical masks or N95 masks should be the preferred choices as they have least impact on speech understanding (9, 48, 55, 56).
- Use of remote microphone technology in educational settings and when communicating with people with hearing loss in order to compensate for the fact that face coverings attenuate signals originating in front of the listener (3).
- Use augmentative strategies to improve communication when wearing a mask. These include speaking slowly and clearly–shouting does not help someone with hearing loss, use gestures, when repeating a statement rephrase rather than repeat verbatim, write down key information for patients who are struggling, and do not assume that the absence of a hearing aid indicates good hearing, or that a nod implies a person has heard and understood what was said (37, 38, 58, 61).
- Consider whether virtual/remote medical consultations in some instances might yield more successful communication than in-person masked consultations (12, 44, 65) especially if published guidelines for communication during phone and video consultations are followed (66–69). Furthermore, HCPs should counsel people with hearing loss about the value of live subtitling in virtual communications/video calls to further improve their speech understanding (44).

### Limitations

Although this review was conducted following a search of three databases, gray literature and studies that were not published in English were omitted, therefore, some relevant work might have been missed. Furthermore, given the nature of scoping review, a critical appraisal of studies was not undertaken and therefore we do not comment on the quality of the included studies. As the majority of the included studies were published during the COVID-19 pandemic, rapid measures without baseline measures were conducted, often online/virtually. Therefore, most studies included in this review used survey measures while randomized clinical trial studies were limited. Interpreting findings from survey studies should be highly evidence based, and limitations associated with surveys should be acknowledged, e.g., recall accuracy, sampling bias etc. Future studies should consider planning and execution of studies to accommodate studies of stronger design to produce higher level of evidence (e.g., pre- vs. post-test design). There is a need for further research and development of transparent masks with improved acoustic characteristics to help eliminate the trade-off between clarity of sound and access to visual cues. In addition, studies considering the effect of amplification on speech understanding difficulties caused by face coverings is limited, therefore future studies should explore this as well as. The impact of preventative measures on communication for people with unilateral hearing loss is unknown. Therefore, future studies should consider including people with unilateral hearing loss, with possible comparisons to people with normal hearing and people with bilateral hearing loss.

## Conclusion

Preventative measures are essential at present, to stem the spread of COVID-19. However, these impact verbal and non-verbal communication in everyday life, with the most substantial effects in healthcare settings and for people with hearing loss. As these measures are likely to be a feature in everyday life for the foreseeable future and as the impacts extend beyond hearing and speech understanding difficulties, the use of carefully selected face coverings should be supplemented with augmentative compensatory strategies to support clear and effective communication. Research is also required for development of masks that have better acoustic characteristics while allowing access to visual cues. This can help mitigate negative psychosocial effects and improve quality of care during the uncertain and stressful COVID-19 times.

## Data Availability Statement

The original contributions presented in the study are included in the article/Supplementary Material, further inquiries can be directed to the corresponding author.

## Author Contributions

IO, GS, VM, and DS: establishment of research question(s), development of search strategy, extraction of primary studies from the included reviews, discussion, and conclusions. DS: background framing. IO and GS: database search, record screening, and lead writers of manuscript. All authors contributed to the design of the work, discussed the results, commented on the manuscript, and approved the manuscript and its submission to Frontiers in Public Health.

## Funding

IO is a post-doctoral fellow at the University of Pretoria supported by a grant from Sonova, AG. GS was supported by NIHR Manchester Biomedical Research Centre.

## Conflict of Interest

GS is an unfunded investigator on EPSRC grant EP/V051571/1 titled Improved face-worn PPE designs for use by the public and professionals to reduce audio-visual communication difficulties. The remaining authors declare that the research was conducted in the absence of any commercial or financial relationships that could be construed as a potential conflict of interest.

## Publisher's Note

All claims expressed in this article are solely those of the authors and do not necessarily represent those of their affiliated organizations, or those of the publisher, the editors and the reviewers. Any product that may be evaluated in this article, or claim that may be made by its manufacturer, is not guaranteed or endorsed by the publisher.
