# Supplementary material for: Impact of SARS-CoV-2 Virus (COVID-19) Preventative Measures on Communication: A Scoping Review
Source: Front Public Health. 2022 Mar 28;10:815259. doi: 10.3389/fpubh.2022.815259 (PMC8995421; doi:10.3389/fpubh.2022.815259)
Supplement: Supplementary file 2 [file Table_2.DOCX]

Supplementary Material

Supplementary Table 2a: Summary of key findings of studies relating to subcategory 1a: Impacts of PPE and/or distancing on communication in healthcare context: HCP-HCP communication

| **Authors** | **Aim(s) relating to preventative measures and communication** | **Study sample description** | **Data collection methods** | **Data collected relevant to communication** | **Summary of findings** | **Conclusion** |
| --- | --- | --- | --- | --- | --- | --- |
| Benítez et al. (2020)^22^ | To assess surgeons’ perceptions of the impact of PPE on ability to conduct emergency surgery | 125 surgeons and 9 surgical trainees from 26 countries. | Survey (online) | Opinions about conducting surgery while wearing PPE | 54% reported PPE caused communication issues: N95/ FFP2 respirators muffled speech, FSs attenuated sound | Face coverings disrupt communication in the operating room |
| Duan et al. (2021)^23^  *Also see Table 2b* | To investigate the how PPE affected HCPs during the COVID-19 pandemic | 386 HCPs, 104 of whom worked directly with COVID-19 patients | Survey (online) | Duration of daily mask wear, type of PPE, discomfort experienced, reported negative impacts on performance | 19% reported communication obstacles due to PPE. | Communication and other problems associated with wearing PPE in clinical settings must be solved |
| Eddy (2021)^24^  *Also see Table 2b* | To explore how NHS employees perceive the effects of PPE and social distancing on social aspects of care | 464 employees of a NHS mental health service | Survey (online) | Perceived social impacts of COVID-19 within the work environment | Majority said face masks and social distancing had detrimental effect on communication and rapport. 62% said masks negatively impacted interaction and communication with colleagues. | PPE and social distancing negatively impact communication and rapport between colleagues |
| Foula et al. (2021)^25^ | To examine effect of PPE on physician communication | 272 physicians (54 in emergency medicine; 120 surgeons; 98 other) | Cross-sectional survey (online) | Reported negative effects of PPE on communication | 75.5% said communication was negatively affected by PPE, with a further 14.1% saying communication was sometimes affected. | PPE affects communication between physicians regardless of medical specialties |
| Grote et al. (2021)^26^ | To determine how the communication, confidence at work and well-being of D/deaf HCPs were impacted by  opaque masks. | 83 health professionals with HL (31 different professions were represented with 18 doctors and 14 nurses). 68% had severe or profound HL; 57% wore HAs, 15% wore CIs | Survey (online) | Hearing and communication preferences, access to workplace assistive technology, access to transparent masks, impacts of masks on communication | 89% reported masks made communication with (patients and) colleagues ‘harder’ or ‘impossible’,  > 50% said mask-related communication difficulties made them anxious at work, affected their wellbeing and made them worry about making mistakes. 17% were removed from patient-facing roles due to mask-related communication difficulties. Most felt the communication needs of D/deaf HCPs had not been met during the pandemic. | Opaque masks have had multiple adverse effects on D/deaf HCPs |
| Hayirli et al. (2021)^30^ | To categorize and describe barriers to teamwork posed by PPE and distancing in the emergency setting. | 55 professionals in an emergency department (18 nurses, 17 physicians, 7 nurse practitioners and physician assistants, 5 pharmacists, 3 social workers) | Semi-structured interviews (online) | Experiences with teamwork and communication before and during the COVID-19 pandemic, and how the pandemic affected work | PPE led to material and barriers. Material barriers (masks) disrupted communication, and interpersonal relationships, muffled information flow, impeded facial recognition, and reduced belonging and cohesion, and increased interpersonal strain. Spatial barriers (distancing, limiting the number of people in a room) resulted in mediated communication between team members. | Both material and spatial barriers affected communication and HCP-HCP relationships |
| Hignett et al. (2020)^27^ | To understand how PPE changed conduct of clinical tasks | 405 responses from NHS staff (292 women, 111 men). | Survey (online) | Questions about  fit and comfort of PPE, reading, operating equipment, hearing, communicating, reaching, moving, and dexterity for different types of PPE | Surgical masks and visors led to problems communicating, and hearing alarms, etc., Women reported more difficulty than men. | PPE impacts HCP-HCP communication.  The design of PPE must be improved to overcome human factor/ergonomic issues. |
| Hoernke et al. (2021)^31^ | To report on frontline healthcare workers’ (HCWs) experiences, fears and concerns with PPE and its perceived impact on ability to deliver care. | 46 HCWs (majority were doctors and nurses working in hospital settings) | Semi-structured telephone interviews with HCWs (n=46) plus review of newspaper articles (n=39), social media posts (n=145000) and government PPE policies (n=25) | Interviews: Perceptions and experiences with PPE during COVID-19 pandemic. | PPE made it more difficult to recognize colleagues. | Use of PPE results in  physical discomfort, practical problems, and communication barriers for HCPs. |
| Parush et al. (2020)^28^ | To examine opinions about  physical, ergonomic, perceptual, and cognitive factors  associated with use of PPE | HCPs involved in the care of COVID-19 patients: 722 from Israel and 301 from Portugal | Survey (online) | Ratings of PPE for physical, ergonomic, perceptual, and cognitive factors | Most HCPs reported PPE cause difficulty hearing and understanding speech. Increased PPE discomfort was associated with greater communication difficulty, both of which were associated with to reduced situational awareness. | PPE negatively impacts HCPs’ vision, hearing, speech understanding, situational awareness, and decision making |
| Singh et al. (2021)^29^ | To assess discomforts associated with use of PPE during surgery | 220 clinicians from 13 countries in gynecologic oncology practice. 95% were aged between 35-45 years | Survey (online) | Details about PPE usage and associated discomfort | Most common discomfort associated with the use masks was communication difficulty in the operating room | PPE causes communication difficulties and discomfort when performing surgery. |

Note: Note: reference numbers are added in the first column for cross-referencing

PPE = personal protective equipment, FFP2 = filtering face mask (equivalent to N95 respirator), N95 = N95 respirator, FS = face shield, HCP = healthcare professional, NHS = National Health Service, HL = hearing loss, HA = hearing aid, CI = cochlear implant, HCW = healthcare worker

Supplementary Table 2b: Summary of key findings of studies relating to subcategory 1b: Impacts of PPE and/or distancing on communication in healthcare context: HCP-patient communication

| **Authors** | **Aim(s) relating to preventative measures and communication** | **Study sample description** | **Data collection method** | **Data collected**  **relevant to communication** | **Summary of findings relevant to communication** | **Conclusion** |
| --- | --- | --- | --- | --- | --- | --- |
| Deardorff et al. (2021)^32^ | To understand HCPs perspectives regarding effect of PPE on communication in healthcare settings | 257 HCPs | Survey (online) | Awareness of HL since COVID-19, impacts of HL on care, and how this is managed | Relative to before the pandemic, 25% more HCPs reported patient HL affected quality of care. ~40 % said communication issues had increased since the start of the pandemic. This was attributed to mask wearing (37.1%) and longer encounters (20%). Solution included HAs, communication strategies, writing things down and sound amplifiers. Captioning and speech-to-text apps were rarely used. | Health systems need new processes and interventions to ensure people with HL receive equitable health care |
| Eddy (2021)^24^ *Also see Table 2a* | To explore how NHS employees perceive the effects of PPE and social distancing on social aspects of care | 464 employees of a NHS mental health service | Survey (online) | Perceived social impacts of COVID-19 within the work environment | 63% said social cohesion between employees and patients had been negatively affected, 39% agreed that the ability to attend to patients’ social needs had been adversely impacted. 65% said masks negatively impacted interactions with service users. | PPE and social distancing perceived to negatively impacts interactions and communication between HCPs and patients |
| Ferrari et al. (2021)^38^ | To explore the impact of PPE and social distancing on communication and relationship between nurses, caregivers and children | 17 nurses and 17 caregivers | Semi-structured interviews (in-person) | Caregiver and nurse perceptions of the impact of PPE and social distancing on communication and  relations in the hospital setting | PPE and distancing led to communication problems between nurses, children and care givers and consequent frustration among nurses, inability to grasp cues from facial expressions, caregiver inability to lipread, and children being fearful of nurses wearing PPE.  To overcome problems nurses spoke more loudly, clearly and slowly, repeated content and simplified content, and used gestures more intensively. | PPE and social distancing create barriers to effective communication |
| Gaucher et al. (2021)^33^ | To explore Canadian emergency physicians’ experiences, concerns, and perspectives during the first wave of the COVID-19 pandemic | 187 emergency physicians (51% female, age: 30-69 years) | Survey (online) | Changes in staff–patient–family interactions and pandemic institutional dynamics and communication  strategies | 97% said PPE changed patient care leading to difficulties creating a bond with patients, limited non-verbal communication and facial expressions, barriers to verbal communication, fear in pediatric patients, and decreased time needed to don and doff PPE. | Emergency physicians identify that patient interaction is affected due to the use of PPE |
| Gopichan-dran & Sakthivel (2021)^34^ | To assess how COVID-19 preventive measures impact patient trust in their HCPs. | 359 adults attending a tertiary care hospital | Survey (on handheld device at care center). | HCP-patient communication, and patient trust in HCPs. | > 60% reported communication difficulties due to physical distance, masks, PPE. This affected patients’ understanding of instructions given by HCPs. There was a negative correlation between HCP-patient communication barriers and trust in HCPs | COVID-19 preventative measures impact HCP-patient communication and may compromise trust. |
| Grote et al. (2021)^26^  *Also see Table 2a* | To determine how the communication, confidence at work and well-being of D/deaf HCPs were impacted by  opaque masks | 83 health professionals with HL (31 different professions were represented with 18 doctors and 14 nurses) 68% had severe or profound HL; 57% wore HAs, 15% wore CIs | Survey (online) | Hearing and communication preferences, access to workplace assistive technology, access to TMs, impacts of masks on communication | 89% reported masks made communication with patients ‘harder’ or ‘impossible’, 30% reported patients would remove their masks to facilitate communication with them. | Opaque masks have adverse effects on D/deaf HCP-patient communication |
| Hoernke et al. (2021)^31^  *Also see Table 2a* | To report on frontline healthcare workers’ (HCWs) experiences, fears and concerns with PPE and its perceived impact on ability to deliver care. | 46 HCWs (majority were doctors and nurses working in hospital settings) | Semi-structured telephone interviews with HCWs (n=46) plus review of newspaper articles (n=39), social media posts (n=145000) and government PPE policies (n=25). | Interviews: Perceptions and experiences with PPE during COVID-19 pandemic. | PPE made it more difficult to build rapport with patients. Particular problems arose with older patients and people who relied on lipreading. In some situations (e.g. breaking bad news, obtaining consent) masks were removed | PPE impacts HCP-patient interpersonal relationships and rapport. This is particularly so for older people and people with HL. |
| Imai & Furukawa (2021)^35^ | To investigate the impacts of masks and plastic partitions on patient-HCP communication and subjective anxiety among patients with psychiatric disorders | 425 adult patients visiting a psychiatric clinic. (Mean age = 53.1 (SD: 14.7) years. 60.9% had stress-related conditions, 26.8 had mood disorders, 10% psychoses. | Survey (completed at clinic) | Effect of masks/plastic partitions on patient willingness to describe emotions and thoughts to the HCP, COVID-19 anxiety | Masks and plastic partitions were not barriers to communication and did not change willingness to discuss emotions and thoughts with HCP but they did decrease COVID-19 anxiety | Masks and plastic barriers in psychiatric clinics are helpful for reducing patient anxiety and don't disrupt willingness to communicate |
| Kraztke et al. (2021)^36^ | Randomized trial to evaluate patients' perceptions of TMs vs opaque masks on communication during surgical clinic encounters. | 200 patients (114 women, 86 men; mean age = 54.5 years) rating 15 surgeons (6 female, 9 male) randomized to wear a TM or opaque mask during surgery | Survey  (clinicians: paper and electronic; patients: virtual) | Patients: Perception of surgeon communication, and empathy, impression of mask, trust in surgeon. Surgeons: likelihood of future use of a TM | Patients’ perspective:  Patients rated surgeons more positively on understandability of explanations (TM 95% vs opaque 78%), demonstration of empathy (TM 99% vs opaque 85%), trust (TM 94% vs opaque 72%). Patients preferred TMs citing improved understanding of communication and appreciation for visibility of the face. Surgeons’ perspective: Surgeons did not rate TMs highly, >50% were unlikely to use one in future, citing concerns about appropriate protection. Some were willing to use a TM if patient benefit can be demonstrated. | Patients prefer to see their surgeon’s face and find this advantageous for communication, empathy and trust. Surgeons do not trust that TMs give adequate protection. |
| Shack et al. (2020)^37^ | To assess how masks impact engagement with pediatric patients, and to find solutions to overcome this. | 356 Pediatric HCPs | Survey (online) | Effect of masks on ability to engage with and assess patients, and compensation strategies. | 82% said masks disrupt interaction with patients. 63% said children were fearful of HCPs wearing masks. 59% reported difficulties assessing or treating patients when wearing a mask. Effects were greatest with youngest patients (< 2yr.). More experienced HCPs had less difficulty. Non-verbal communication, removal of mask and TMs were used to overcome problems. | The use of face masks affects interaction and engagement between HCPs and pediatric patients. |

Note: reference numbers are added in the first column for cross-referencing

HCP = healthcare professional, PPE = personal protective equipment, HL = hearing loss, HA = hearing aid, CI = cochlear implant, NHS = National Health Service, HCW = healthcare worker, TM = transparent mask

Supplementary Table 3a: Summary of key findings of studies relating to subcategory 2a: Impacts of face coverings and/or distancing on communication in everyday life for the general public

| **Authors** | **Aim(s) relating to preventative measures and communication** | **Study sample description** | **Data collection methods** | **Data collected** | **Summary of findings** | **Conclusion** |
| --- | --- | --- | --- | --- | --- | --- |
| Carbon (2020)^7^ | To examine impact of masks on emotion recognition as it pertains to communication | 41 individuals age 18-87 (mean 26.7 yr.) | Behavioral testing (online) | Recognition of emotions and confidence ratings for 144 face stimuli: 6 emotions (anger, disgust, fear, happiness, neutral, and sadness) x gender (male/female) x age of face (young, middle age, elderly) × 2 individuals x mask condition (no mask/mask) | Emotion recognition 90% accurate without mask. Significantly lower accuracy for recognition of anger, disgust, happiness and sadness with mask. Significantly lower confidence ratings for recognition of all emotions. | Face masks may complicate social interaction as they disrupt ability to recognize emotions from facial expressions. |
| Carbon & Serrano (2021)^39^ | To examine impact of masks on emotion recognition among school-age children as it pertains to everyday communication and the classroom | 57 school children (aged 9-11 yr., mean = 9.7 yr.; 28 female, 29 male) | Behavioral testing (online) | Recognition of emotions for 48 face stimuli: 6 emotions (anger, disgust, fear, happiness, neutral, and sadness) x gender (male/female) x age of face (young, middle aged) × mask condition (no mask/surgical mask) | Emotion recognition ~90% accurate without mask. Significantly lower accuracy for recognition of disgust, fear, and sadness with mask. Significantly higher accuracy for recognition of anger and neutral states with mask. | Masks may affect children's reading of emotions from facial expressions - which is important for affirmation and thus healthy social and psychological development. |
| Cheok et al. (2021)^41^ | To evaluate public perceptions of, attitudes towards, and experiences with masks | 402 individuals (patients and accompanying persons) attending an outpatient clinic (age 15-98, mean 54 yr., SD 16 yr.) | Survey (completed at clinic) | Duration of mask wear per day, type of mask worn, perceived necessity or wearing a mask, and discomfort experienced | 46.5% reported communication difficulty when wearing a mask, independent of age, gender and mask type. | Masks cause discomforts yet in Singapore, the public is compliant with their use. |
| Coniam (2005)^40^ | To examine the effect of wearing a face mask on oral test scores with and without testers and students wearing masks | 186 students in Grade 11 and 16 raters trained in test assessment | Behavioral testing and student ratings. Self-ratings of raters and interviews with a subset of 4 raters. | Students took oral tests and rated their own performance, their speaking style, and how well they could understand their classmates in masked and unmasked conditions,  Raters rated students’ performance while masked and unmasked, and perceptions of, and reactions, to masks | Test scores were not impacted by masks although self-rated performance was lower, as were ratings of the understandability of class mates. Students said they spoke more slowly and loudly when wearing a mask and that communication was harder because they couldn't see facial expressions. Raters' data were mixed with some feeling masks were detrimental and others feeling it had had no effect on test scores. | The impacts of masks should be considered during oral testing because while it masks do not impact measured performance, they impacted self-rated performance and speaking style. |
| Kastendieck et al. (2021)^8^ | To assess whether masks impact emotion recognition, interpersonal closeness, and affiliative facial mimicry and how the this interacts with visual context | 200 members of UK general public (mean age 32.9 yr., SD 12.7 yr.) | Behavioral testing (online) | Participant's facial expressions were videoed while viewing video clips of faces in 48 conditions: emotion (happy/sad) x gender (male/female) x 3 actors x masking (mask/no mask) x background scene (store/countryside). Each participant saw half the complete set. | Mask had no effect on emotion recognition. Masks reduced perceived emotion intensity, perceived interpersonal closeness and facial mimicry for happiness. There was no effect of visual scene. | Face masks complicate social interactions and lack of mimicry can impact social bonding in positive situations. |
| Malzanni et al. (2021)^42^ | To determine the impact of masks and social distancing on communication, mental health, and perception of general health. | 200 adults with confirmed normal hearing (Mean age = 40.5 yr.), 137 had a public-facing job, 63 did not. | Survey (online) | Ratings of impact of masks and safety distance on communication. | Use of face masks was related to a decrease in quality of life. Face masks cause more communication difficulties than social distancing. This was attributed to sound attenuation and lack of ability to see facial expressions. | Face masks and social distancing affects communication and general health |
| Saunders et al. (2020)^12^  *Also see Table 3b* | To understand the impact of face coverings on hearing and communication when wearing a mask and when communicating with someone wearing a mask. | 460 members of UK general public | Online survey | Self-reported hearing, ratings of impact of masks on ability to hear, understand, feel engaged and feel connect with someone wearing a mask. Open-ended comments regarding impacts of masks on communication | Masks impacted rated ability to hear, understand, feel engaged and feel connected in a conversation regardless of whether hearing difficulties were reported. Impacts were greater when communicating in a healthcare context than when communicating with family/friends or at work. Masks impacted hearing, use of visual cues, content of interactions, psychosocial state, approaches to communication, and social behaviours. | Masks disrupt communication. and cause anxiety and stress, especially in medical settings. |

Note: reference numbers are added in the first column for cross-referencing

SD = standard deviation, UK = United Kingdom

Supplementary Table 3b: Summary of key findings of studies relating to subcategory 2b: Impacts of face coverings and/or distancing on communication in everyday life for people with hearing loss

| **Authors** | **Aim(s) relating to preventative measures and communication** | **Study sample description** | **Data collected** | **Summary of findings** | **Conclusion** |
| --- | --- | --- | --- | --- | --- |
| Abrar et al. (2021)^43^ | To explore the impact of COVID-19 and postponement of elective CI implantation surgical procedures on emotional well-being and everyday life for profoundly deaf patients. | 23 adults with profound HL whose scheduled CI implantation surgery was postponed due to COVID-19 (aged 18-89 yr., mean = 69 yr.) | Open-ended questions on impact of postponement of CI surgery on emotional well-being and daily life; impact of COVID-19 on emotional well-being and daily life. | Surgery delay had negative impacts on well-being, including feelings of loneliness and isolation. Older participants reported low mood, depression, and hopelessness; younger participants reported frustration and anxiety. Participants reported face masks caused difficulties communicating and that social distancing led to the need for more phone conversations - which were difficult. Some reported increased reliance on family members for activities of daily living and loss of personal independence. Difficulties were experienced with the use of online communication platforms in education and employment. | Postponement of CI implantation surgery negatively impact mental well-being and everyday life for people with profound HL as COVID-19 preventative measures exacerbate the challenges faced due to hearing loss. |
| Dunn et al. (2020)^47^ | To understand from an auditory ecology and psychosocial perspective how social isolation during COVID-19 impacted CI users | 48 adult CI users with at least 12 months CI experience | Self-reported listening experiences, situations, and outcomes measured pre-COVID and during COVID (ecological momentary assessment and surveys) | Social distancing resulted in less diverse and more favorable listening conditions, better speech understanding, reduced listening effort, and fewer activity limitations and less social isolation due to hearing loss. | Spending more time at home during COVID resulted in quieter environments leading to fewer communication challenges for CI users. |
| Gaeta (2020)^46^ | To explore older adults’ perceptions of their hearing health during the COVID-19 stay-at-home order | 150 adults (100 women, 50 men; mean age = 74.4 yr., SD = 6.34, range: 60–90) who were members of lifelong learning organizations for older adults. About 50% wore HAs | Self-reported hearing, use of hearing assistive technology, likelihood of visiting an audiologist during COVID-19, impacts of masks on wearing of HAs and communication, opinions about teleaudiology. | Most were open to teleaudiology appointments and felt quality of care would be unchanged, although in-person care was preferred. >50% reported wearing HAs with a mask was problematic, and that they had experienced difficulty communicating when a mask was worn. Most said their likelihood of going to their audiologist hadn’t changed as a result of COVID restrictions. | Audiologists should consider telepractice or remote service delivery options to meet the needs of their patients. |
| Naylor et al. (2020)^44^ | To explore the perceived effects of social distancing and masks on the scope and extent of hearing-related difficulties encountered in everyday life by people with HL | 129 adults (aged 27-79 years, mean = 64.4 years) with hearing loss (confirmed) living in Scotland. 55% wore 2 HAs, 19.4% wore one HA. | Audiometric and self-reported hearing, HA ownership and use, impacts of COVID lockdown restrictions on behavior, emotional state, hearing, and tinnitus.  Data were analyzed by self-reported hearing: Better Hearing (BH; self-rated hearing ‘very good’ ‘good’ or ‘middling’) and Worse Hearing (WH, self-rated hearing ‘poor’ or ‘very poor’). | BH group used their HAs less during lockdown. WH group reported increased anxiety when communicating with people wearing masks or from a distance, reported more difficulties communicating in video calls than in person, and had concerns about access to audiology services. They also felt some relief at not being obliged to attend social gatherings in difficult listening situations. Almost all participants agreed that masks muffled speech and made communication harder because the mouth was not visible and thought key workers should be supplied with transparent masks. There was some indication that face mask strings interfere with wearing HAs. | The worse the hearing, the greater are the negative impacts of social distancing and masks.  Lifestyle changes were positive for some people because they encountered fewer difficult listening situations. |
| Saunders et al. (2020)^12^ *Also see Table 3a* | To understand the impact of face coverings on hearing and communication when wearing a mask and when communicating with someone wearing a mask. | 460 members of UK general public, ~40% rated their hearing as ‘poor’ or ‘very poor’ ~50% used HAs or other hearing assistive technology | Self-reported hearing, ratings of impact of masks on ability to hear, understand, feel engaged and feel connect with someone wearing a mask. Open-ended comments regarding impacts of masks on communication | Masks impacted the content of communication, feelings of interpersonal connection and willingness to engage in conversation, resulted in increased anxiety and stress, and decreased self-confidence, and made communication fatiguing,  frustrating and embarrassing. These impacts were greater for people with HL than for those without. | Masks disrupt communication. They have greater impacts for people with HL and in important communication situations |
| Schafer et al. (2021)^45^ | To document potential difficulties encountered by students with HL during the pandemic | 416 respondents (38% educational audiologists, 41% teachers of deaf/HoH, and other educators) | Questions about mask wearing and their impacts on children with and without HL in the classroom, and about accommodations provided | Remote or blended learning was common. Students and caregivers had some problems with remote learning technology. In the classroom, students had difficulty hearing teachers wearing opaque masks and social distancing increased these difficulties. >75% of teachers and students wore transparent masks always or sometimes. Seating arrangements were usually adjusted to accommodate students with hearing loss, sign language interpreters were provided as needed. | Additional accommodations for students with HL are required for remote learning |

Note: reference numbers are added in the first column for cross-referencing

CI = cochlear implant, HL = hearing loss, HA = hearing aid, SD = standard deviation, UK = United Kingdom, HoH = hard of hearing

Supplementary Table 4a: Summary of key findings of studies relating to subcategory 3a: Impacts of face coverings (i.e., face masks, face shields etc.) on speech assessed via acoustic measures

| **Authors** | **Aim(s) relating to preventative measures and communication** | **Types of face covering evaluated** | **Data collection methods. Acoustic measures reported here** | **Data collected** | **Summary of findings** | **Conclusion** |
| --- | --- | --- | --- | --- | --- | --- |
| Atcherson et al. (2021)^15^ | To examine the acoustic impacts of different face coverings | NM, SM x 2, respirator masks x 2, carbon filter mask, homemade FM x 2, TM x 4, shield-type mask, FS x 3 | Acoustic analysis | Acoustic attenuation (in dB) relative to the no mask condition, total RMS values, directional effects in 15 degree increments at 3ft and 6ft distance | All face coverings attenuated sounds >1kHz. TMs attenuated sound more than other masks tested, SMs attenuated sound the least. Directional impacts varied by mask, with FSs having greater forward attenuation and deflecting sound towards sides and back. Impacts of TMs were dependent on size of plastic window and distance between the plastic window and the wearer's mouth. | TMs attenuate sound more than opaque masks; the effect is dependent on individual differences in size of the transparent window and position on the face |
| Atcherson et al. (2017)^6^  *Also see Table 4b* | To evaluate measure acoustic effects of masks and effect of hearing status and mask type on speech recognition performance. | NM, SM, TM | Acoustic analysis, behavioral testing | Total root mean square (RMS) values for NM, SM, and TM. | RMS values highest for NM and lowest for TM. | Masks attenuate the acoustic signal, TMs have greater impact than SM. |
| Bottalico et al. (2020)^48^  *Also see Table 4b* | To explore the effects of wearing face masks on classroom communication | SM, FM, N95 | Acoustic analysis, behavioral testing | Attenuation (dB/octave). | Attenuation greatest at >2kHz. FM was most attenuating, SM the least | SMs are less attenuating than masks made from other materials. |
| Caniato et al. (2021)^49^ | To understand to how various masks impact speech intelligibility, sound level and directivity within a classroom | SM, SM+ transparent panel, SM+FS, FM x 4, FM+FS, FFP2, FS | Acoustic analyses | Speech transmission index (STI) assessed at various locations in two simulated classrooms of differing RTs, male and female speakers, and 10 mask conditions | Most masks decreased the STI regardless of RT in all positions tested. Impacts were more negative for male speakers and in the less reverberant room. SMs with or without a transparent window did not impact STI. FSs are most detrimental to STI and directivity | SMs with a transparent panel should be the face covering of choice in the classroom. |
| Corey et al. (2020)^3^ | To examine the acoustic attenuation caused by different face masks | SM, N95, KN95, FM x 6, FM with transparent panel x 2, FS | Acoustic analysis | Acoustic attenuation and directive attenuation of speech for 12 conditions supplemented with microphones to assess how masks impact assistive listening systems | Masks had little effect below 1 kHz. At higher frequencies, SM, N95 and KN95 were least attenuating, TMs and FSs were most attenuating. FMs varied considerably depending on material, weave and fit. All masks attenuated signals from front more than from other directions. FS amplified sound behind the talker. Lapel microphones and microphones on forehead had least effect on signal. FS distorted speech for all microphone placements. | Masks attenuate  sounds in front of the talker above 4kHz. TMs and FSs are most attenuating but to allow visual cues and high-frequency signals a TM mask combined with a lapel microphone is effective. |
| Fiorella et al. (2021)^50^ | To investigate the impact of SMs on voice and verbal communication | NM, SM | Vocal analysis | Acoustical properties of voice (F0, intensity, jitter, shimmer, and harmonics-to-noise ratio) for sustained /a/ spoken by 60 individuals (mean age 47 yr., range 26-69; 24 male, 36 female). | 65% of participants showed a decrease in vocal intensity when wearing a SM relative to NM. No effects on any other parameters. | Surgical masks do not significantly alter the vocal signal |
| Homans & Vroegop (2021)^51^  *Also see Table 4b* | To investigate the effect of SMs and FSs on speech perception of adults with moderate to severe hearing loss. | NM, SM, FS | Acoustic analysis,  behavioral testing | Acoustic attenuation | Face shield attenuates high frequencies more than SM. | FSs impact the acoustic signal more than SMs. |
| Magee et al. (2020)^52^  *Also see Table 4b* | To evaluate the impact of masks on acoustic analysis, voice analysis, speaking rate and perceived intelligibility of speech | NM, SM, FM, N95 | Acoustic analysis, behavioral testing | Measures of timing, frequency, perturbation, and power spectral density in for spoken speech: sustained /a/ and passage reading. | N95 attenuated frequencies >3kHz; SM and FM attenuated frequencies >5 kHz. FM and N95 resulted in more pauses during speaking relative to NM, leading to slower speech rate. | Masks change the speech signal, but some specific acoustic features remain largely unaffected (e.g., measures of voice quality) irrespective of mask type. |
| McKenna et al. (2021)^53^ | To examine the effect of mask-wearing on speech acoustics and self-reported vocal symptoms in HCPs | SM, N95, N95+SM | Acoustic analysis; self-reported vocal effort and dyspnea (breathing difficulty) | Acoustic analysis: acoustic Vowel Articulation Index (VAI), cepstral and spectral acoustic measures,  Vocal measures: relative fundamental frequency (RFF) | Masks reduced VAI, high-frequency information, and RFF offset 10 and cause increased cepstral peak prominence and perceived vocal effort. High-frequency attenuation was greatest for N95 mask | Face masks impacts the speaker by altering speech acoustics, and thus the requirement to compensate with increased vocal effort. |
| Mendel et al. (2008)^13^  *Also see Table 4b* | To investigate the effects of SMs on speech spectrum and speech perception for individuals with normal hearing and hearing loss. | NM, SM | Acoustic analysis, behavioral testing | Total root mean square (RMS) values for NM and SM. | RMS values higher for NM than for SM. | SM affected acoustics relative to NM |
| Muzzi et al. (2021)^54^  *Also see Table 4b* | To assess the effect of common types and combinations of F-PPE (face personal protective equipment) on speech intelligibility in quiet and in noise. | NM, SM, FS, SM+FS, FFP2 x 3, FFP2+SM x 2, FFP2+FS x 3. | Acoustic analysis; behavioral testing | Spectral analysis and Speech Intelligibility Index (SII) | PPE conditions had little/no effect at frequencies <1000 Hz, but attenuated frequencies >1000 Hz. FFP3+FS caused greatest high frequency attenuation and reduced SII by up to 25% in noise conditions. | PPE impacts the speech signal, FFP2+FS is most detrimental. |
| Palmiero et al. (2016)^9^ | To evaluate the impact of facial PPE on speech intelligibility using the STI | Protective face mask (like a SM), N95, elastomeric half-mask air-purifying respirator (EAPR). | Acoustic analysis | STI measured for a modified version of the National Fire Protection Association 1981 standard in noise and pink noise at 2 levels. | STI decrement over NM:  Protective face mask: 3-4%, M95: 13-17%, EAPR: up to 45% | The use of PPE reduces speech intelligibility in noise. PF (similar to SM) has the least effect on speech intelligibility in noisy situations. |
| Thibodeau et al. (2021)^14^  *Also see Table 4b* | To assess the effect of face mask type on auditory-visual speech recognition in noise for adults with normal and hearing loss and to determine if differences were due to visual cues or to acoustic factors | NM, TM, TM with transparent panel covered with fabric | Acoustic analysis, behavioral testing (online) | Acoustic attenuation | Maximum attenuation for TM and TM with fabric cover relative to NM was at 4-5kHz, value were 11.3 dB and 13/6 dB respectively. | TM with or without the plastic visible attenuate speech at higher frequencies. |
| Toscano & Toscano (2021)^55^  *Also see Table 4b* | To assess the effects of face masks on acoustics and speech recognition performance in noise. | NM, SM, FM (fitted), FM (pleated), N95 respirator | Acoustic analysis, behavioral testing (online) | Spectral analysis, acoustic energy | Masks primarily attenuated high frequencies (above 2 kHz); the FM were most attenuating, SM had little impact on the acoustic signal. | SM has the least effect on the acoustic signal |
| Vos et al. (2021)^56^  *Also see Table 4b* | To measure the effects of face coverings on acoustic speech cues, and speech recognition performance of cochlear implant (CI) users | NM, N95, N95+FS, TM x 2 | Acoustic analysis, behavioral testing | Spectral analysis | All face coverings attenuated high frequencies. TMs masks were more attenuating than N9. The N95+FS was most attenuating (17.3 dB at 5 kHz). | N95-FS greatly attenuates high frequencies |

Note: reference numbers are added in the first column for cross-referencing

NM = no mask; SM = surgical mask, TM = transparent mask; FM = fabric mask, FFP2 = filtering face mask (equivalent to N95 respirator), N95 = N95 respirator, FS = face shield; RT = reverberation time, VM=ventilated mask, STI = speech transmission index, HCP = healthcare provider

Supplementary Table 4b: Summary of key findings of studies relating to subcategory 3b: Impacts of face coverings (i.e., face masks, face shields etc.) on speech assessed via behavioral measures

| **Authors** | **Aim(s) relating to preventative measures and communication** | **Study sample description** | **Data collection methods. Behavioral measures reported here** | **Data collected** | **Summary of findings** | **Conclusion** |
| --- | --- | --- | --- | --- | --- | --- |
| Atcherson et al. (2017)^6^  *Also see Table 4a* | To evaluate the effect of hearing status and mask type on speech recognition performance in noise (SiN) under auditory only (A) and audio-visual (AV) conditions | 30 adults, 10 per group: NH (age 19-64 yr., mean= 28.5 yr.); Moderate hearing loss (HL; PTA 41-70 dB HL; age 20-74 yr., mean = 49.6yr.); Severe HL (PTA > 70 dB HL, age 22-68 yr., mean = 48.7 yr.) with NM, SM, TM | Acoustic analysis, behavioral testing | Percent correct rationalized arcsine units for NM-A, NM-AV, TM-A, TM-AV and SM-A. | SiN performance of NH listeners not impacted by masks or A vs. AV conditions. People with moderate HL performed better in NM-AV and TM-AV than SM-A and TM-A. People with severe HL performed better in NM-AV and TM-AV than NM-A, SM-A and TM-A. | Masks impact speech perception in noise. People with HL benefit from visual cues provided by a TM, even though the acoustic signal is poorer than with NM or a SM. |
| Bandaru et al. (2020)^57^ | To assess the impact of PPE on speech  understanding in quiet | 20 healthcare workers with NH (PTA<25 dB HL, age = 23-54 yr., mean = 40 yr.). | Behavioral testing | SRT, SDS for live voice testing in quiet with NM and N95+FS | Significantly poorer SRT (mean decrement of 12.4dB) and SDS (mean decrease of 7%) for N95+FS relative to NM | Speech understanding is significantly impacted by PPE |
| Bottalico et al. (2020)^48^  *Also see Table 4a* | To explore the effects of wearing face masks on communication in college classrooms | 40 English-speaking college students | Acoustic analysis, behavioral testing (online) | Percent correct word recognition for mask (SM, N95 and FM) x reverberation times (short, long) presented at +3dB SNR; rating of listening effort after each condition. | Significant effects of masks and room conditions with poorest performance with FM. Speech was 12% poorer with the SM, 13% poorer with N95, and 16% poorer with FM.  Listening effort with masks was greater than with NM. No difference in listening effort between masks. | Masks impact speech perception in noise in classroom settings |
| Cohn et al. (2021)^58^ | To examine whether mask wearing differentially impacts comprehension of speech when it is spoken in differing styles. | 63 native speakers of English age 18-25 yr. (mean = 20 yr., SD = 1.4). | Behavioral testing (online) | Word identification in noise in 6 conditions: masking (NM, FM) x speech styles (casual, emotional, clear) x speakers (male/female) | No main effect of masking. Interaction between masking and speech style such that performance was better for (a) masked clear speech than non-masked clear speech and (b) non-masked emotional speech than masked emotional speech. | Speakers change how they speak to compensate for masks for clear speech. |
| Giovanelli et al. (2021)^59^ | To examine the impact of face masks on speech comprehension during video calls | 36 participants with self-reported NH (mean age 26.0 yr., age range = 19–40 yr.; 30 females, 6 males) | Behavioral testing (online simulating a video call with multiple talkers) | SiN performance, confidence and listening effort scores, and analysis comparing measured and perceived performance for A, AV, AV+SM | Performance, confidence and measured vs. perceived accuracy were poorer, and effort was greater for A and AV+SM than AV. | Limited visual cues resulted in poor speech perception, confidence and perceived listening effort. |
| Hampton et al. (2020)^60^ | To identify measurable challenges to speech discrimination in noise whilst wearing PPE | 5 individuals at ENT department (3 men and 2 women; age range = 29–49 yr., median age = 39 yr.) | Behavioral testing | SiN performance in simulated hospital conditions (office, emergency room, intensive care unit, operating room) x masking (NM, FFP3+FS) x speech level (normal, raised) | FFP3+FS affected speech performance in simulated environments with high levels of noise (operating room, intensive care unit). Raised speech level improved performance | PPE impacts communication in healthcare environments, raising voice level can overcome problems but  may lead to vocal strain. |
| Homans & Vroegop (2021)^51^  *Also see Table 4a* | To investigate the effect of SMs and FSs on speech perception of adults with moderate to severe hearing loss | 42 patients with moderate to severe hearing loss (aged 31-85 yr., man = 65 yr.); 23 CI users, 19 HA users. | Acoustic analysis, behavioral testing | Speech perception in quiet and speech tracking (words/minute) for NM, SM and FS. | Participants performed better when they could see the face (NM and FS) than when it was covered (SM). The poorer the hearing the worse the impacts of the SM. | People with moderate to severe HL benefit from visual cues even when acoustic attenuation is greater |
| Magee et al. (2020)^52^  *Also see Table 4a* | To evaluate the impact of masks on acoustic analysis, voice analysis, speaking rate and perceived intelligibility of speech | Seven adults (aged 21-39 yr., mean = 28.1 yr., 3 female, 4 male) | Acoustic analysis, behavioral testing | Rated intelligibility and accuracy of spoken speech assessed in quiet for NM, SM, FM, N95 | Masks did not significantly influence perceived intelligibility for words or sentences; however, accuracy of word and sentence identification was affected by all masks similarly | Face masks change the accuracy of spoken words and sentences. |
| Mendel et al. (2008)^13^  *Also see Table 4a* | To investigate the effects of SMs on speech spectrum and speech perception for individuals with normal hearing and hearing loss. | 30 adults: 15 with NH (aged 22 to 54 yr., mean = 28 yr.); 15 with HL (PTA mean = 39.3 dB HL; aged 23 to 75 yr., mean = 58 yr.) | Acoustic analysis, behavioral testing | Percent correct scores converted  to rationalized arcsine units for  masking (NM, SM), x condition (quiet, noise) | SM did not impact performance in noise for either listener group. Both group performance more poorly in noise than in quiet. | SM did not affect speech perception in noise. |
| Muzzi et al. (2021)^54^  *Also see Table 4a* | To assess the effect of common types and combinations of F-PPE (face personal protective equipment) on speech intelligibility in quiet and in noise. | 10 adults with NH tested | Acoustic analysis, behavioral testing | Speech recognition in noise (% correct) measured 12 mask conditions: NM, SM, FS, SM+FS, FFP2 x 3, FFP2+SM x 2, FFP2+FS x 3. | Percent correct score decreased for all masks. Decrement was least for SM (23.3%) and most by FFP3 ventilated mask+face shield (69.0%) . | The use of PPE impairs communication with the most negative effect caused by combinations of ventilated face mask + face shield. |
| Smiljanic et al. (2021)^61^ | To understand masks affect intelligibility  and memory for native and non-native speech | 250 students who were native English speakers (mean age = 23.2 yr., SD = 5.0 yr.) | Behavioral testing (online) | AV word recognition and story recall for speakers (Native English, native Spanish) x masking (NM, SM) x speech style (clear, conversational) x SNR (+5, 0, -5 dB) | SM did not affect word recognition or memory for either speaker in quiet. In noise, SM decreased word recognition and recall of conversational speech relative to no mask. | Masks impact speech understanding and recall in noise but not in quiet |
| Thibodeau et al. (2021)^14^  *Also see Table 4a* | To assess the effect of face mask type on auditory-visual speech recognition in noise for adults with normal and hearing loss and to determine if differences were due to visual cues or to acoustic factors | Study 1. 154 participants (mean age = 43 yr., 11 males, 143 females), 133 with NH, 10 with HL who use hearing assistive technology (AT) and 11 with HL who do not use AT.  Study 2. 29 participants (mean age = 25 yr, 3 males, 26 females). | Acoustic analysis, behavioral testing (online) | Study 1. SiN-AV and ratings of confidence and concentration for NM, TM, and TM with transparent panel covered with fabric.  Study 2. SiN-A for TM, and TM with transparent panel covered with fabric | Performance was best in NM condition and better in Av condition with TM than TM with transparent panel covered with fabric. The converse was true in the A-only condition. Confidence rating followed the same pattern as performance. Participants with HL who used AT reported less confidence and increased concentration compared to the other 2 groups. | TM benefits were attributable to the addition of visual cues. TMs are beneficial for people with HL and NH. |
| Toscano & Toscano (2021)^55^  *Also see Table 4a* | To assess the effects of face masks on acoustics and speech recognition performance in noise. | 181 participants with self-reported NH (mean age = 37 yr.) | Acoustic analysis, behavioral testing (online) | Percent correct speech recognition for SNR (+3, +13) x masking (NM, SM, FM-fitted, FM-pleated, N95 x talker (Male, female) | Masks had no impact at +13 dB SNR. At +3 SNR the FM and N95 masks were detrimental to performance, SM was not. | Masks affect performance when background noise is greater |
| Vos et al. (2021)^56^  *Also see Table 4b* | To measure the effects of face coverings on acoustic speech cues, and speech recognition performance of cochlear implant (CI) users | 23 adult CI users with at least 6 months of device use (14 females, 9 males) | Acoustic analysis, behavioral testing (online) | Aided speech recognition performance in quiet for NM, N95, N95+FS | Participants performed similarly in NM and N95 conditions but performed significantly worse in N95+FS condition. | N95 mask alone did not affect speech recognition of CI users, the addition of a FS had a significant detrimental effect. |

Note: reference numbers are added in the first column for cross-referencing

PTA = pure tone average, SiN = speech in noise, NM = no mask; SM = surgical mask, TM = transparent mask; FM = fabric mask, FFP2 = filtering face mask (equivalent to N95 respirator), FFP3 = filtering face mask code 3, FS = face shield; RT = reverberation time, VM=ventilated mask, STI = speech transmission index, HCP = healthcare professional, A = auditory only testing, AV = audio-visual testing, HL = hearing loss, NH = normal hearing, SRT = Speech reception threshold, SDS = speech discrimination score, SNR = signal to noise ratio
